# Contrasting Patterns of Raccoon (*Procyon lotor*) Spatial Population Genomics Throughout a Rabies Management Area in Eastern North America

**DOI:** 10.1111/eva.70105

**Published:** 2025-05-12

**Authors:** Matthew W. Hopken, Clara P. Mankowski, Christine Thurber, Antoinette J. Piaggio, Kathleen M. Nelson, Richard B. Chipman, Zaid Abdo, Tore Buchanan, Ariane Massé, Amy T. Gilbert

**Affiliations:** ^1^ US Department of Agriculture, Animal and Plant Health Inspection Service, Wildlife Services, National Wildlife Research Center Fort Collins Colorado USA; ^2^ Department of Microbiology, Immunology, and Pathology Colorado State University Fort Collins Colorado USA; ^3^ US Department of Agriculture, Animal and Plant Health Inspection Service, Wildlife Services, National Rabies Management Program Concord New Hampshire USA; ^4^ Wildlife Research and Monitoring Section, Ontario Ministry of Natural Resources, 2140 East Bank Dr., Trent University Peterborough Ontario Canada; ^5^ Ministère de l'Environnement, de la Lutte Contre les Changements Climatiques, de la Faune et des Parcs, Gouvernement du Québec Québec Canada

**Keywords:** dispersal, microhaplotypes, rabies, spatial autocorrelation, wildlife disease, zoonotic disease

## Abstract

Wide‐ranging, generalist species provide both interesting and challenging opportunities for research questions focused on population structure. Their continuous distributions and ability to occupy diverse habitat types can obscure genetic signals of ancestry and geographic clustering. However, spatially informed population genetic approaches are notable for high‐resolution identification of geographic clusters that often elude more classical clustering models. The northern raccoon ( *Procyon lotor* ) is a broadly distributed species in North America, with populations in diverse habitats ranging from dense urban to rural landscapes. Wildlife management agencies have an interest in understanding raccoon ecology, given their propensity for human‐wildlife conflicts and zoonotic diseases. We combined samples from an extensive raccoon tissue repository with a RADcapture panel of 1000 microhaplotype loci to conduct spatial genetic analyses of raccoon populations in eastern North America. Our objective was to estimate patterns of genetic diversity on the landscape that may inform raccoon rabies management. Bayesian clustering analyses delineated multiple ancestry clusters that encompassed large areas across 22 US states and 2 Canadian provinces. We discovered a potential phylogeographic split between central and southern samples from those in the northeast region, which correlates with post‐Pleistocene recolonization detected in a multitude of species from the region. A finer scale structure was identified using spatially explicit analyses and demonstrated variable dispersal/gene flow patterns within specific regions. The Appalachian Mountain region restricted local connectivity among raccoons, while raccoon populations in central New York, the Ohio River Valley, southern Québec, and southern Alabama demonstrated high genetic connectivity. The results from this study highlight how raccoon ecology and historical biogeography can help contextualize contrasting hypotheses about the influence of landscape on raccoon movement patterns, which can inform management of zoonotic disease risks at regional scales.

## 1 Introduction

The northern raccoon ( *Procyon lotor* Linnaeus, 1758) is a highly adaptable species and one of the most broadly distributed North American mammals, with its native range encompassing Central America to central Canada (Zeveloff 2002). Historically, raccoons were most abundant in eastern North America and rare in the western United States (US). Following a decline of raccoons in the early twentieth century, the populations have rebounded and expanded to 49 US states and the western Canadian provinces (Zeveloff 2002). The species can thrive in a seemingly unlimited variety of habitats, including human‐dominated landscapes (Hadidian et al. 2010). This adaptability has facilitated the incidental establishment of populations outside mainland North America, including multiple Caribbean Islands, Europe, and central and eastern Asia (Farashi et al. 2013; Helgen et al. 2008; Ikeda et al. 2004; Louppe et al. 2019; Salgado 2018). From both ecological and evolutionary perspectives, the raccoon provides opportunities to test many hypotheses about historical biogeography, rapid ecological adaptation, and animal responses to human activities. One aspect of raccoon ecology that has been studied mostly at local scales but remains less understood on a larger scale is dispersal and how it shapes both historical and contemporary distributions of the species. The research that does exist has led to confounding results about population structure, gene flow, and the geographic distribution of phylogenetic lineages.

Cullingham, Kyle, et al. (2008) conducted a broad raccoon phylogenetic study using mitochondrial DNA to test hypotheses about the consistency of the genetic and morphological descriptions of subspecies in the US and Canada. Similar to earlier studies with allozymes, regional groups demonstrated genetic homogeneity, and population genetic variation generally did not correlate with subspecific taxonomy based on morphologic data (Cullingham, Kyle, et al. 2008; Dew and Kennedy 1980; Hamilton and Kennedy 1987; Kennedy and Lindsay 1984). However, some historical biogeographic signals were detectable in the phylogenetic data, such as the multiple times Florida was isolated from the mainland during the Pleistocene (Cullingham, Kyle, et al. 2008). Local‐scale studies have investigated raccoon population structure and gene flow patterns associated with landscape and habitat features with varying results. Rivers and topographic features, such as hills and valleys, were not associated with raccoon population structure, but distance, habitat fragmentation, and resource availability were correlated (Côté et al. 2012; Dharmarajan et al. 2009; Johnson et al. 2009; Rioux Paquette et al. 2014; Root et al. 2009). The most influential features impacting raccoon genetic divergence have been impassable water features such as the Niagara River and channels between islands and the mainland (Cullingham et al. 2009; Moncrief et al. 2017; Trujillo and Hoffman 2017). In a recent study using high‐throughput sequencing (HTS) data, Hopken et al. (2023) evaluated raccoon population structure in the northeastern US and found hierarchical structuring with large genetic clusters connected through isolation‐by‐distance (IBD). However, there has been no follow‐up investigation of raccoon population genetic structure across the geographic range represented in Cullingham, Kyle, et al. (2008) using HTS data from the nuclear genome.

Continuously distributed and broad‐ranging species that are habitat generalists provide an informative backdrop to understand how ecological and evolutionary factors have shaped regional biodiversity (Caro 2010). Because these species are neither imperiled nor have experienced drastic reductions in effective population sizes, they maintain historic signals of evolutionary changes in their genomes and can be proxies for understanding both historical and contemporary population connectivity to inform management and conservation approaches that account for both prior and current ecological conditions (Hopken et al. 2013). Of particular interest is elucidating zoonotic pathogen transmission pathways through an understanding of how continuously distributed reservoir species are connected across a region, which can inform management strategies to limit health risks to humans and animals.

Genetic approaches have informed wildlife management for decades through the identification of population and management units that inform targeted strategies to meet management objectives (Moritz 1994). In wildlife diseases, pathogen maintenance and attenuation have been directly linked to non‐random contact between individuals and populations of a host species; thus, understanding spatial structure, gene flow, and dispersal of the host species can help inform transmission pathways (Biek and Real 2010; Blanchong et al. 2016; Messinger and Ostling 2009). A pertinent example of genetics informing wildlife disease management at the landscape scale is tracking the source of multi‐host pathogen spread in sympatric species. Vander Wal et al. (2013) evaluated the population structure of two cervid species susceptible to *Mycobacterium bovis* . Through population genetic and epidemiological data, they determined that the spread of the pathogen among the two species was likely facilitated by dispersing white‐tailed deer ( *Odocoileus virginianus* ). The authors further argued for better incorporation of evolutionary approaches in wildlife disease management due to the multitude of questions that can be addressed with the methods (e.g., host‐pathogen‐environment dynamics; Van der Wal et al. 2014). Continuously distributed generalist species, however, pose challenges to commonly used population genetics analyses as the species often lack discrete population boundaries (assumptions of many analyses) even though dispersal distance is limited (Frantz et al. 2009; Schwartz and Mckelvey 2009). Spatial population genetics can help disentangle cryptic structure and spatial autocorrelation in systems where IBD may be the predominant mechanism impacting population connectivity and pathogen transmission (Frantz et al. 2009; Meirmans 2012).

In this study, we use an HTS microhaplotype genotyping panel designed for raccoons and a repository containing thousands of tissue samples to evaluate the population genetic structure and gene flow patterns of raccoons in eastern North America. Consistent with prior studies, we expected patterns of IBD and used both non‐spatial and spatial clustering analyses. We utilized spatial autocorrelation analyses to highlight both contemporary and historical processes that shaped raccoon population structure and then provide perspective on the management of raccoon diseases with a particular focus on the control and local elimination of *Lyssavirus rabies* raccoon variant in North America.

## 2 Materials and Methods

### 2.1 Samples

Raccoon tissue samples for this study were a combination of samples previously analyzed in Hopken et al. (2023) and newly genotyped samples. The new samples were collected opportunistically during routine enhanced rabies surveillance and oral rabies vaccination monitoring activities from 2018 to 2022. Ear tissue samples were collected in silica beads from specimens and were stored at −80°C upon receipt from the field. Genomic DNA was extracted from tissues using the DNeasy Blood & Tissue Kit (Qiagen, Germantown, Maryland, US) following the Tissues and Rodent Tails protocol on a QIAcube (Qiagen). The DNA concentration of extracts was measured using a Qubit 4 Fluorometer (Thermo Fisher Scientific, Waltham, Massachusetts, US).

### 2.2 Sequencing and Bioinformatics

Each sample was genotyped following a custom microhaplotype panel and bioinformatic approach designed for raccoons (Hopken et al. 2023). Briefly, the genotyping protocol was based on the RADcapture approach of Ali et al. (2015) and MyBaits hybridization probes (Daicel Arbor Biosciences, Ann Arbor, Michigan, US) that targeted approximately 1000 independent loci from raccoons. Following library preparation, the newly collected samples were sequenced as 150‐base pair (bp) reads on a Miseq or Nextseq 2000 (Illumina, San Diego, CA, US). Bioinformatic processing included demultiplexing and PCR clone removal using stacks2 (Rochette et al. 2019), quality trimming and filtering using trimmomatic v0.39 (Bolger et al. 2014), mapping reads to microhaplotypes using bwa v0.7.17 (Li and Durbin 2009), and microhaplotypes were genotyped using the R package microhaplot (Baetscher et al. 2018; https://github.com/ngthomas/microhaplot). We removed any individual raccoon genotype that had > 0.30 missing data using poppr v2.9.4 in R v4.1.3 (Kamvar et al. 2015; R Core Team 2022; Kamvar et al. 2014). Sequence data are available from the NCBI Sequence Read Archive (accession numbers: PRJNA950910 and PRJNA1149082).

### 2.3 Bayesian Cluster Analysis

To estimate the number of clusters and assign individual raccoons to the clusters, we used the Bayesian population clustering algorithm implemented in the software structure v2.3.4 (Pritchard et al. 2000). For datasets with low‐frequency minor alleles, the software manual recommends estimating the allele frequency prior (λ) rather than running the default value of 1. We used the estimated value of λ = 0.47 from Hopken et al. (2023). We then ran the algorithm with a burn‐in of 250,000 and MCMC length of 500,000 for *K* = 1–20 with 20 replicates at each *K*. Given that multiple *K* values can represent population structure in continuously distributed species with low genetic divergence, we looked for correspondence among a combination of *K* estimators which included *ΔK* and the Puechamaille estimators MedMedK, MedMeanK, MaxMedK, and MaxMeanK calculated using Structureselector (Evanno et al. 2005; Li and Liu 2018; Puechmaille 2016). The Puechamaille estimators help identify spurious cluster assignments based on the median and mean ancestry coefficient Q of the cluster, and any cluster that falls below the set median and mean is removed. This results in genetic cluster estimates that are often less than the raw estimate of *K* from structure, which has been shown through simulations to match the true number of clusters in the dataset (Puechmaille 2016; Stankiewicz et al. 2022). The Q‐matrix from the clumpak algorithm implemented in Structureselector was used to generate bar plots in the r package structuRly (Criscuolo and Angelini 2020; Kopelman et al. 2015).

We quantified both expected (*H*_*E*_) and observed (*H*_*O*_) heterozygosity, calculated the inbreeding coefficient (*F*_*IS*_), and tested for Hardy–Weinberg equilibrium (HWE) per locus using R packages adegenet, poppr, and hierfstat. For each structure genetic cluster, we estimated allelic richness using Pegas with all defaults, except with the extrapolation method because of unbalanced cluster sample sizes (Foulley and Ollivier 2006). Heterozygosity, private alleles, *F*_*IS*_, and *F*_*ST*_ (*ϴ*; Weir and Cockerham 1984) among clusters were estimated using adegenet, poppr, and hierfstat. We removed clusters with fewer than 10 individuals from allelic richness estimates and analysis of molecular variance (AMOVA) to prevent small sample sizes from influencing estimates of variation, since greater than 5 individuals with over 500 loci have been shown to be sufficient for precise estimates (Nazareno et al. 2017).

### 2.4 Spatial Autocorrelation Analyses

**FIGURE 1:**
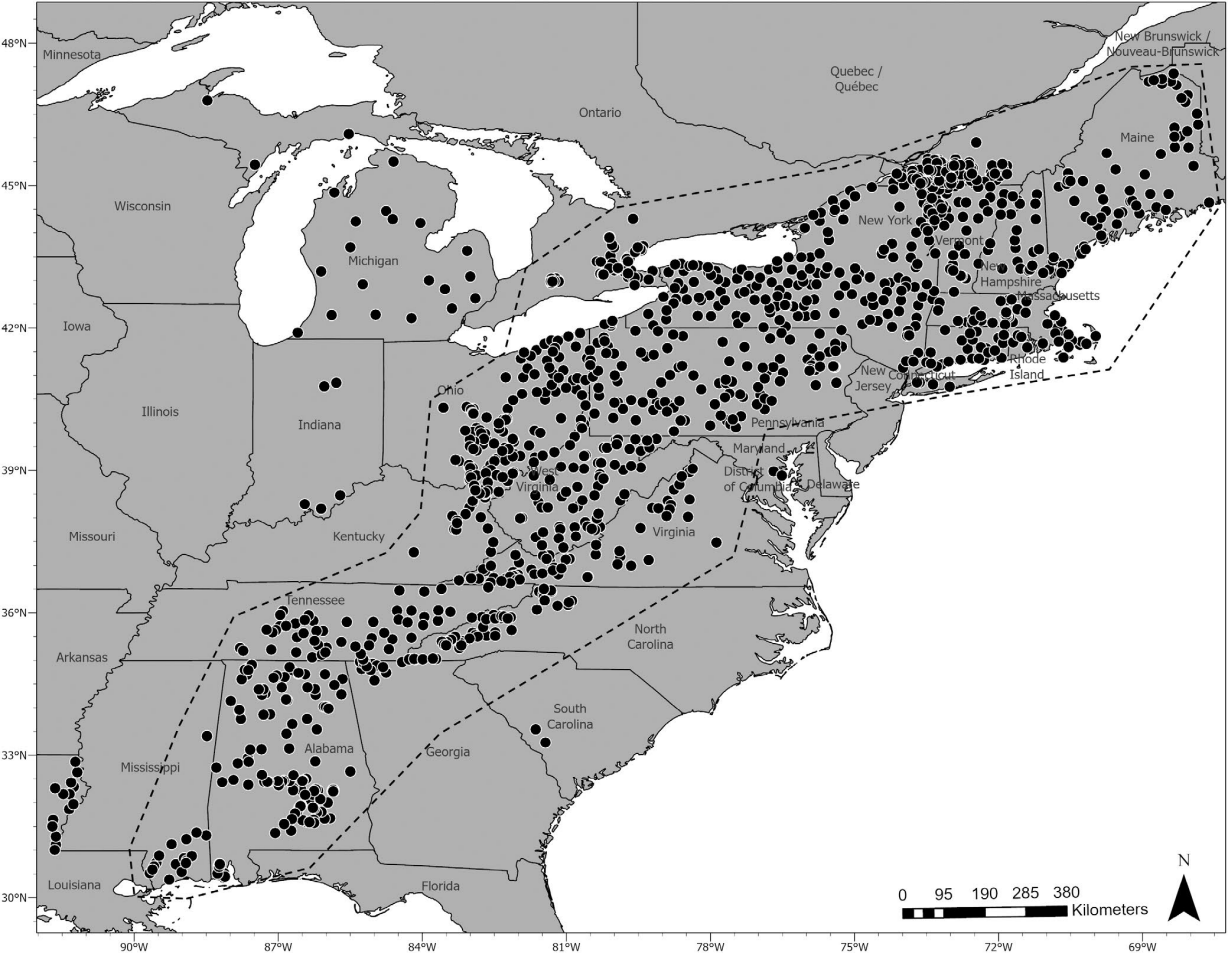
Distribution of raccoon ( *Procyon lotor* ) samples (*n* = 1307) collected across eastern North America from 2018 to 2022. The dashed line specifies samples that were used for spatial autocorrelation analyses.

In continuously distributed species, spatially informed analyses can help tease apart subtle genetic structure (Diniz‐Filho and De Campos Telles 2002). We implemented multiple spatially informed analyses for detecting boundaries and transition zones between clusters to identify geographic clines. We chose to analyze samples that had a relatively continuous distribution to limit the impact of divergent outlier samples. We removed samples from Michigan, Indiana, Louisiana, and South Carolina, as these samples had a geographic gap of approximately 200 km to the nearest samples around managed raccoon populations in eastern North America (Figure 1).

We used spatial principal components analysis (sPCA) implemented in adegenet (Jombart et al. 2008). The sPCA is a spatially explicit ordination that uses Moran's *I* index to identify eigen‐vectors that maximize variation in allele frequencies and spatial autocorrelation. The eigen‐vectors are then mapped onto geographic coordinates to visualize transitions between genetic clusters. This approach has proven powerful for detecting cryptic spatial structure while also being free from population genetic assumptions, such as Hardy–Weinberg and linkage equilibrium. Statistical significance of global structure (positive spatial autocorrelation) vs. local (negative spatial autocorrelation) was assessed using a Monte Carlo test based on multiple regression of Moran's Eigenvector Maps, against a null hypothesis of absence of spatial structure. We generated the spatial network using a Gabriel graph, and the Monte Carlo analysis was run for 1000 permutations.

We also used the R package mapi (Mapping Averaged Pairwise Information) to evaluate spatial autocorrelation and identify potential gene flow barriers using pairwise genetic and geographic distances (Piry et al. 2016). The mapi analysis is robust to the impacts of IBD common in natural populations, including raccoons (Hopken et al. 2023). We first generated variation surfaces from a randomization procedure based on pairwise genetic distances. The variation was then evaluated in a spatial context to determine significantly high or low values in relation to geographic distance between samples to estimate a geographic heatmap of areas with high to low genetic connectivity. Connectivity values in the tails of the distribution were determined to be significant through a nonparametric randomization procedure, corrected for multiple comparisons using false discovery rate. We used Rousset's *ȃ* distance, which accounts for isolation by distance, estimated with spagedi v1.5 (Hardy and Vekemans 2002; Rousset 1999). Individual coordinates were in the Albers Equal‐Area Conic projection, as the mapi package requires Cartesian coordinates. Settings for the algorithm included a spatial dispersion parameter (*β*) of 0.25 for irregular sample distribution, an ellipse eccentricity value of 0.95 for generating pairwise network connections, an error radius around the coordinates of 10 m, and 1000 permutations.

To estimate the geographic extent of spatial autocorrelation, we used a Mantel correlogram among distance classes using the *ȃ* distance and geodesic distance in kilometers to evaluate raccoon genetic distance neighborhoods and to infer differences in localized dispersal and gene flow patterns. The Mantel correlograms included all genotype data used in the mapi and sPCA analyses, and separate tests on samples from five regions that represented high, average, and low spatial autocorrelation identified by the mapi and sPCA analyses (Figure 1). We also used Mantel correlograms to analyze four areas that have had rabies emergency response actions within the last 10 years due to rabid individuals found in raccoon rabies‐free regions. The areas include: Maine; Vermont, New Hampshire, and Québec; Ohio and Pennsylvania; and Alabama, Georgia, and Tennessee. The correlograms were generated using the vegan R package (command mantel.correlog) with 10 km distance classes for the entire dataset, 5 km distance classes for high, low, and average spatial autocorrelations, and 10 km for the four priority rabies management areas (Oksanen et al. 2024). Depending on the habitat and sex of the animal, raccoon home ranges are typically less than 5 km^2^, and dispersal is typically less than 5 km with rare long‐distance movements of less than 100 km (Bozek et al. 2007; Dharmarajan et al. 2009; Gehrt and Frttzell 1997; Hill, Helton, Chipman, et al. 2023; Rosatte et al. 2010). The analysis was run for 1000 permutations, and spatial autocorrelation (both negative and positive) was evaluated using Spearman's correlation coefficient. We used the Holm‐Bonferroni method to correct the significance level for multiple testing (Holm 1979). Distance classes are identified by significant tests and when the *r* ^2^ line crosses from positive to negative values.

## 3 Results

### 3.1 Genotyping

The final data set included 1307 raccoon samples from 22 US states and 2 Canadian provinces genotyped at 1000 microhaplotypes (Figure 1). The mean percent missing data was 9.57%, the mean number of alleles per locus was 4 (range: 2–20), and the mean depth per locus was 188 (range: 59–711). The mean per locus expected heterozygosity was *H*_e_ = 0.32 (range: 0.02–0.8), and the observed heterozygosity was *H*_O_ = 0.27 (range: 0.01–0.79).

### 3.2 Nonspatial Population Structure

**FIGURE 2:**
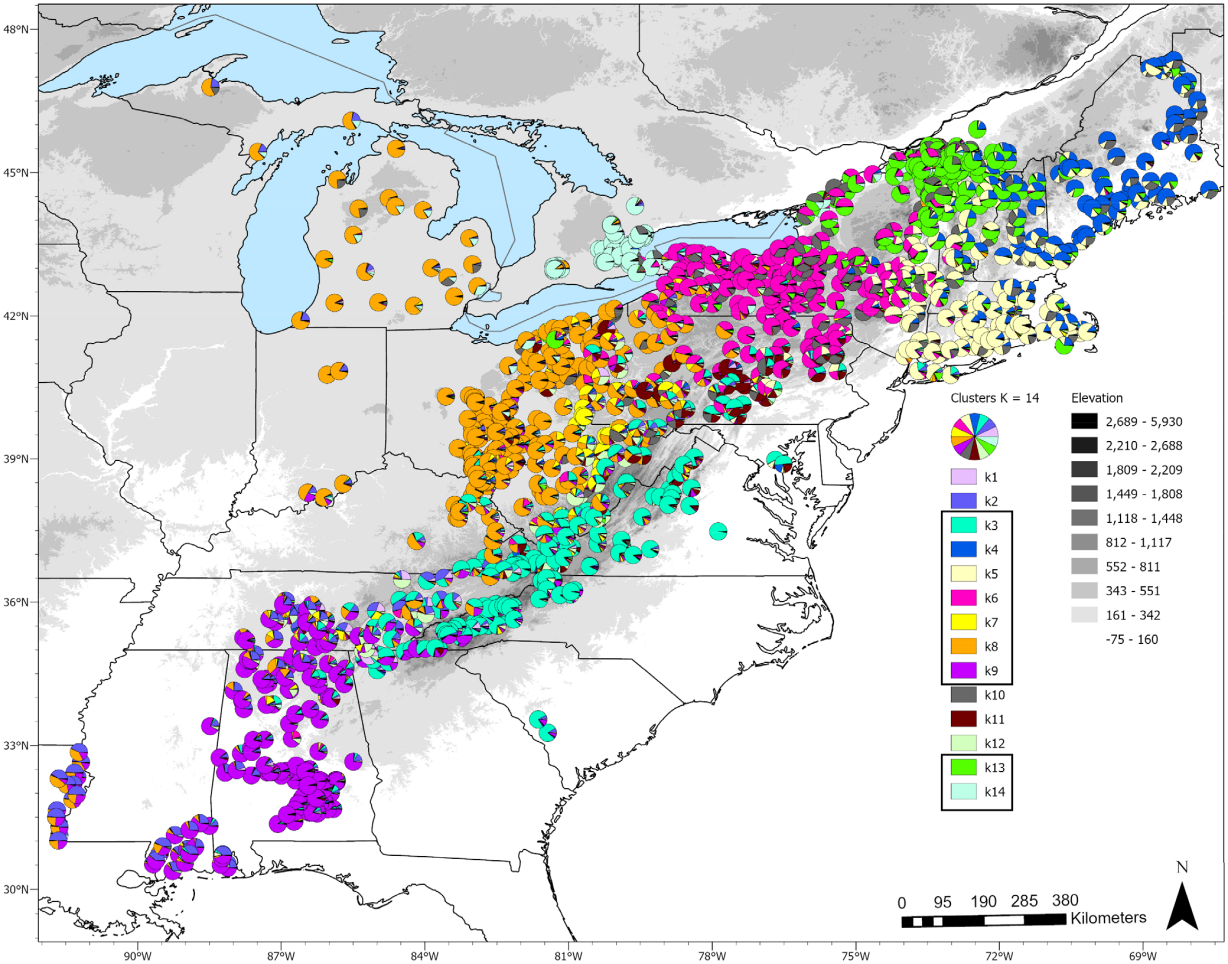
Genetic clustering results from the program structure at *K* = 14 for raccoons from eastern North America. Each pie chart represents an individual raccoon. Pie chart colors represent assignment to a genetic cluster. The boxes around legend items are the nine clusters identified as not spurious by the Puechamaille estimators. Shaded areas represent an elevational gradient in meters.

The structure *K* estimators identified a range of values which included 2, 3,14, and 17 for *ΔK*, and the Puechamaille estimators using the ancestry coefficient of 0.5 plateaued between *K* = 13–15 with an estimated number of genetic clusters of 8–10 after removal of likely spurious clusters (Figures S4 and S5). We focus on *K* = 2 and 14 to represent the highest *ΔK* (*K* = 2) and the estimated *ΔK* that also fell within the range of plateaued *K* values identified by the Puechamaille estimators (Figure S1). Maps of the results for *K* = 2 and 3 are in Figures S2 and S3, while *K* = 14 is in Figure 2. There were 5 clusters with fewer than 10 samples (Table 1). Two of the low sample clusters had maximum ancestry coefficient (*Q*) values less than 0.75. Given the small sample sizes and the low *Q* values of the two clusters, we treated these as spurious assignments in line with the Puechamaille estimators and focused on the nine remaining clusters. The genetic diversity statistics for *K* = 14 are presented in Table 1.

**TABLE 1:** Genetic diversity statistics for populations of raccoons ( *Procyon lotor* ) from eastern North America estimated from 1000 microhaplotypes.

*Cluster K* = 14	Max *Q*	*n*	*H*_*E*_	*H*_*O*_	*P*_*A*_ (283 loci)	*A*_*R*_	*F*_*IS*_
1	0.85	1	—	—	0	—	
2	0.56	2	—	—	0	—	
3	0.96	151	0.31	0.26	47	3.7	0.16
4	0.8	56	0.30	0.27	1	3.4	0.09
5	0.96	96	0.30	0.28	7	3.5	0.09
6	0.96	111	0.31	0.28	6	3.6	0.08
7	0.89	10	0.26	0.11	1	2.6	0.57
8	0.97	221	0.31	0.29	49	3.8	0.09
9	0.98	161	0.30	0.27	60	3.7	0.11
10	0.86	8	0.27	0.25	0	2.6	0.14
11	0.91	8	0.26	0.29	0	2.5	−0.05
12	0.72	6	0.25	0.12	2	2.3	0.50
13	0.97	162	0.31	0.28	5	3.6	0.09
14	0.98	70	0.31	0.30	4	3.5	0.03
Admixed		244	0.32	0.25	40	—	0.20

*Note:* The statistics are sample size (*n*), expected heterozygosity (*H*_*E*_), observed heterozygosity (*H*_*O*_), private alleles (*P*_*A*_) with the number of loci with private alleles in parentheses, allelic richness (*A*_*R*_), and inbreeding coefficient (*F*_*IS*_). Each group of statistics is from clusters identified using a structure with *K* set at 14. The values are means except *P*_*A*_, which is the sum. The sample sizes for the structure clusters only include non‐admixed (*Q* > 0.50) individuals with admixed statistics for all individuals with < 0.5 assignment to any cluster.

The ancestry clusters encompassed large geographic areas that included multiple states and provinces (Figure 2, Figures S2, and S3). At *K* = 2, one cluster spanned the northeastern collection areas and the second spanned populations in the central Appalachian and southeastern US states. The population clusters overlap mostly in central and western New York, Pennsylvania, and Ontario (Figure S1). The nine well‐supported genetic clusters identified from structure at *K* = 14 each covered large areas except K7 and K11, which included 10 or fewer individuals, predominantly located in West Virginia or Pennsylvania, respectively. There were large contact areas between each of the clusters (Figure 2). The cluster with the least amount of admixture was K14, which occurred almost exclusively in Ontario, except for one individual across the Niagara River in New York that was assigned to K6 and K14 (approximately 50% of the genotype assigned to each cluster; Figure 2). There were three individuals in Ontario with partial assignment to the New York cluster K6. Cluster K13 dominated the Champlain Valley in Vermont, northeastern New York, and Québec, with a transition to K4 in samples farther east in Vermont, New Hampshire, and Maine (Figure 2). West of the Champlain Valley, raccoon samples transitioned to K6. Cluster K5 was mostly in Massachusetts, Rhode Island, Connecticut, and southeastern New York. Cluster K3 spanned across West Virginia, Virginia, North Carolina, and northern Georgia, with some partial assignments in Kentucky and Tennessee. The samples in K9 with the highest assignment occurred in southern Alabama. Northern Alabama, central Tennessee, and southern Mississippi, with eastern Louisiana individuals partially assigned to K9 and to the spurious cluster K2.

Genetic divergence (*F*_*ST*_) values between the clusters are provided in Table 2. The average *F*_*ST*_ value is 0.03 (range: 0.01–0.07). The largest value, *F*_*ST*_ = 0.07, is between K4 and K7, with the second highest (*F*_*ST*_ = 0.06) between K5 and K7. The low level of genetic divergence among the clusters is also supported by the AMOVA, with 85.5% of the genetic variation within individual samples (Table 3).

**TABLE 2:** Genetic divergence estimates based on the *F*_*ST*_ estimator *ϴ* for raccoons from eastern North America.

	3	4	5	6	7	8	9	13
4	0.03							
5	0.02	0.02						
6	0.02	0.03	0.02					
7	0.04	0.07	0.06	0.04				
8	0.01	0.04	0.02	0.01	0.04			
9	0.01	0.04	0.03	0.02	0.04	0.01		
13	0.03	0.02	0.02	0.02	0.06	0.02	0.03	
14	0.03	0.05	0.04	0.02	0.05	0.02	0.03	0.03

*Note:* The clusters were defined using the program structure and 1000 microhaplotypes. The clusters in the table are the non‐spurious clusters identified through the Puechamaille estimators.

**TABLE 3:** Analysis of molecular variance (AMOVA) for 14 raccoon genetic clusters in eastern North America.

*K* = 14	Sigma	% variation	φ
Variation between clusters	7.42	2.26	0.02
Variation between samples within clusters	40.27	12.25	0.13
Variation within samples	281.12	85.50	0.15
Total variation	328.80	100.00	

*Note:* The clusters were identified using the program structure and 1000 microhaplotypes.

### 3.3 Spatially Informed Genetic Clines

**FIGURE 3:**
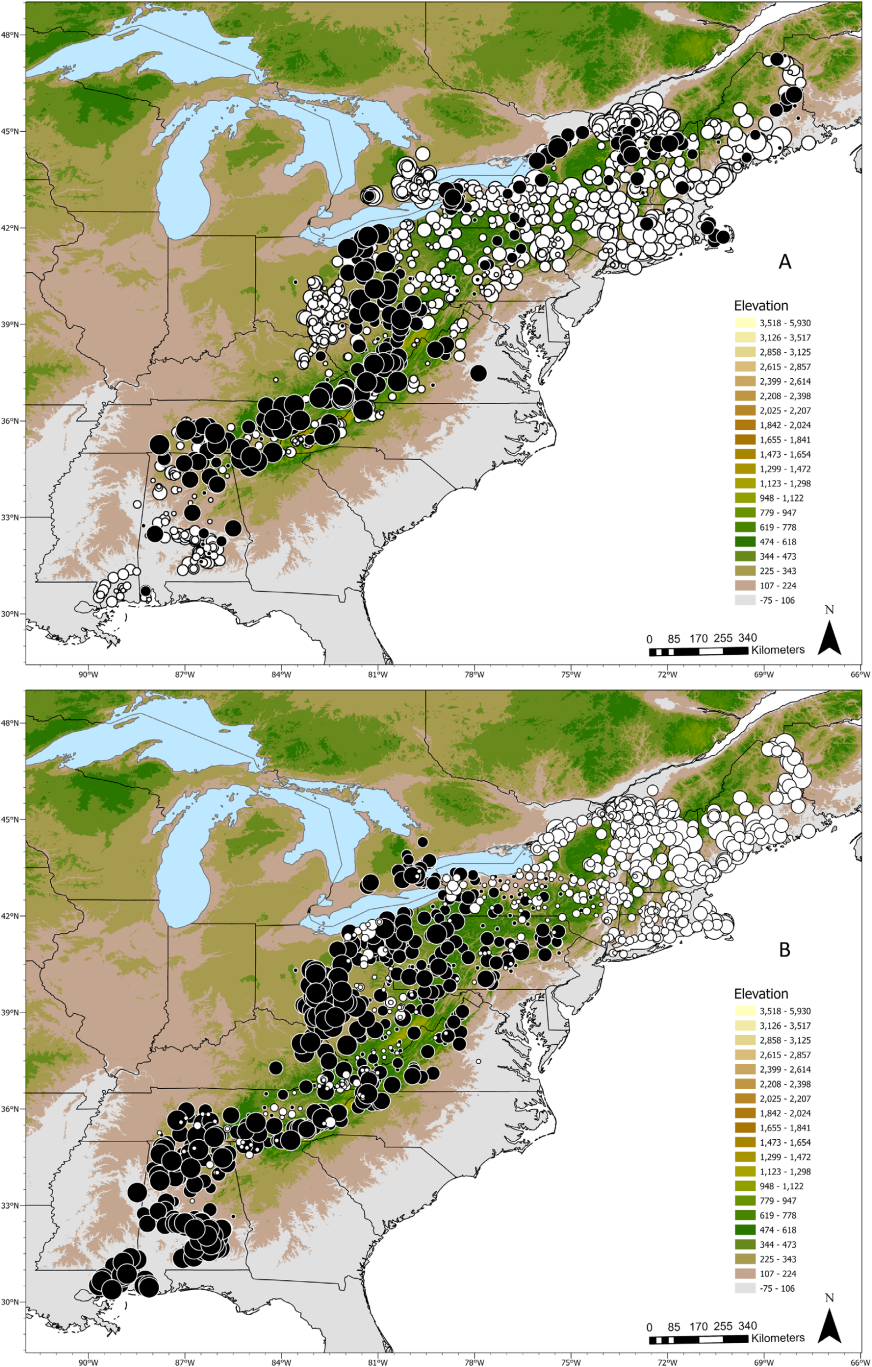
Map of the results from the spatial principal components analysis (sPCA). Each circle is an individual raccoon. Map A shows the result from the first eigen vector, and map B plots the results from the second eigen vector. The colors of the circles represent positive (white) or negative (black) values on the eigenvector. The sizes of the circles denote values closer to zero (small) and further from zero (large). The larger circles are the highest levels of spatial autocorrelation, and black and white samples next to each other represent the transition between genetic groups. The colored shading represents elevation in meters.

Spatial genetic analyses were conducted on 1227 samples after removing data from Michigan, Indiana, Louisiana, and South Carolina. The sPCA identified five eigenvectors that described spatial genetic variation at the global scale and one that was distinct at the local scale (Figure S4). The global (*p* < 0.001) and local (*p* = 0.045) Monte Carlo tests for spatial structure were significant. The bulk of the genetic variation was accounted for in the first decomposed eigenvector (variation = 40.0%; Moran's *I* = 0.52), with the second decomposed eigenvector showing higher spatial autocorrelation but accounted for less of the total genetic variation (variation = 9.7%; Moran's I = 0.85; Figure S4). Eigenvectors 3 and 4 were similar, and 5 and 1225 (local eigenvector) were less informative predictors. Maps of eigenvectors 1 and 2 in Figure 3 demonstrate geographic clustering. Transition zones between genetic groups were similar to the admixture zones identified by the structure analyses (Figure 2). Notable regions with rapid transitions in genetic connectivity were the central and southern Appalachian Mountains, Central Ohio, southern Alabama, and Cape Cod, Massachusetts. The Vermont‐Québec border samples diverged from nearby areas, similar to the structure cluster K13, with a gradual transition of southern Champlain Valley samples northward into Canada. Massachusetts, Connecticut, and Rhode Island diverged from samples north and west, which aligned with structure cluster K5. Ontario raccoons diverged from samples on the east side of the Niagara River in New York. The sPCA eigenvector 2 generally replicated the results of structure at *K* = 2 with two main clusters across the entire sampling area that transitioned in central New York (Figure 3, Figure S1). The main difference was a subset of central Appalachian and Ohio samples that diverged.

**FIGURE 4:**
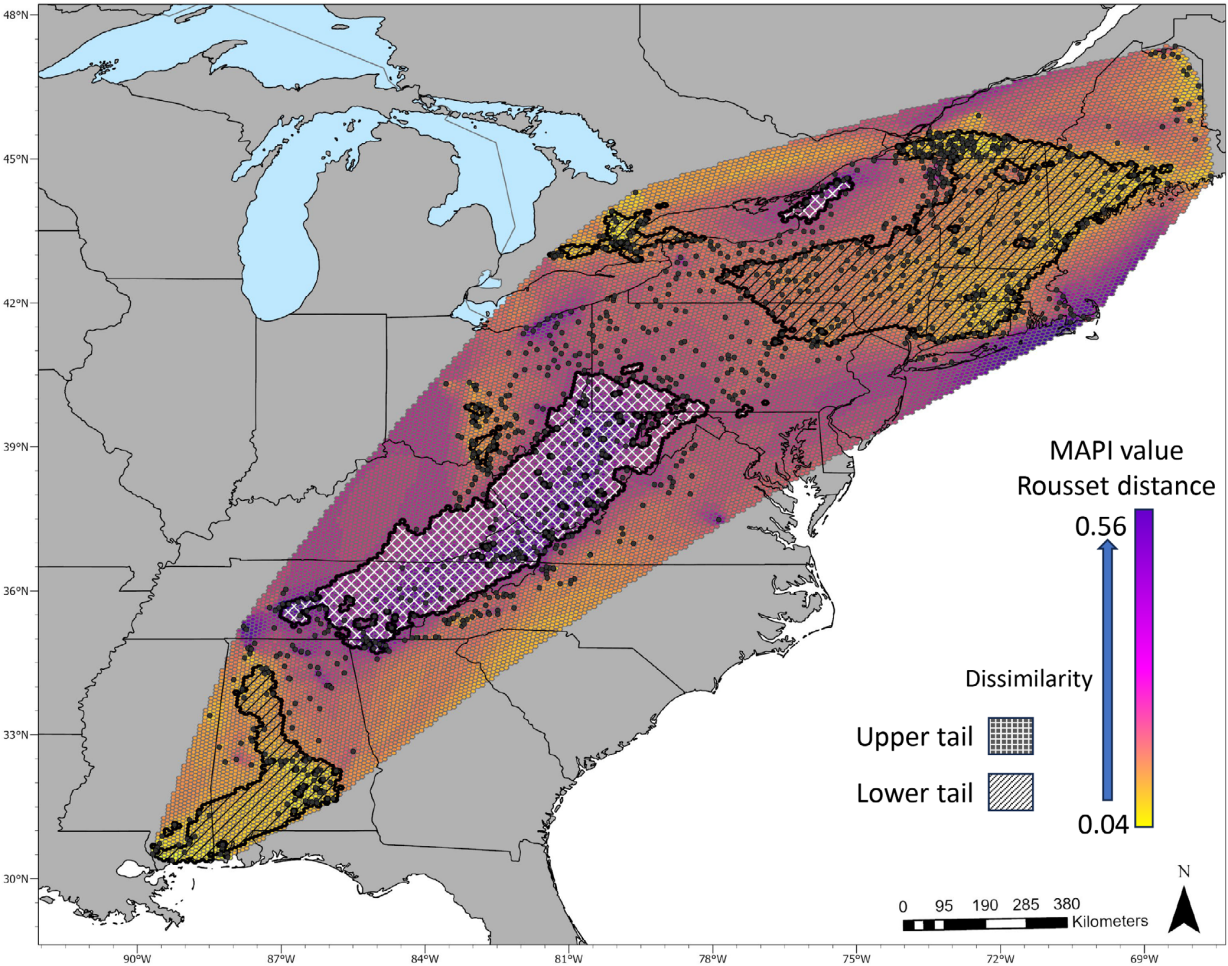
Mapi analysis results as heatmaps overlayed on a map of the study area. The black dots represent sample locations. The colors of the heat map are cooler (purple) for higher dissimilarity and warmer (yellow/orange) for higher similarity. The regions outlined with white cross‐hatching are statistically significant regions of spatial autocorrelation (high discontinuity), and the regions outlined with diagonal lines are significantly low spatial autocorrelation (high connectivity).

The mapi results largely mirrored the sPCA, and to a certain extent, the structure analyses (Figure 4) in supporting large areas of raccoon population connectivity and gene flow. The most significant region of high genetic discontinuity was detected in the central and southern Appalachian Mountains (Figure 4). Cape Cod, Massachusetts; eastern Ohio; and eastern New York–Ontario border were areas with higher levels of discontinuity, but not statistically significant, suggesting reduced animal movement through these areas. In line with the sPCA, central Ohio, northeastern states, and Québec, Ontario around Toronto, and southern Alabama raccoon populations exhibited high connectivity. The mapi analysis provided support for reduced raccoon population genetic continuity between the Champlain Valley in Vermont and surrounding landscapes in Vermont and Québec.

**FIGURE 5:**
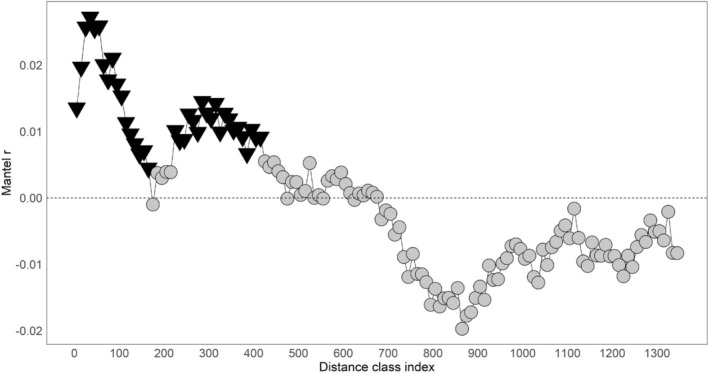
Mantel correlogram for the dataset of 1227 raccoons ranging from Alabama, US, to Ontario and Québec, Canada. The analysis was conducted on Rousset's distance *ȃ* versus geodesic distance. The *x*‐axis represents the distance class indexes for every 10 km (index is centered on the middle value of each class). The *y*‐axis is the Mantel *r* (correlation coefficient). Non‐significant values are represented by gray circles, and statistically significant values are inverted black triangles.

**FIGURE 6:**
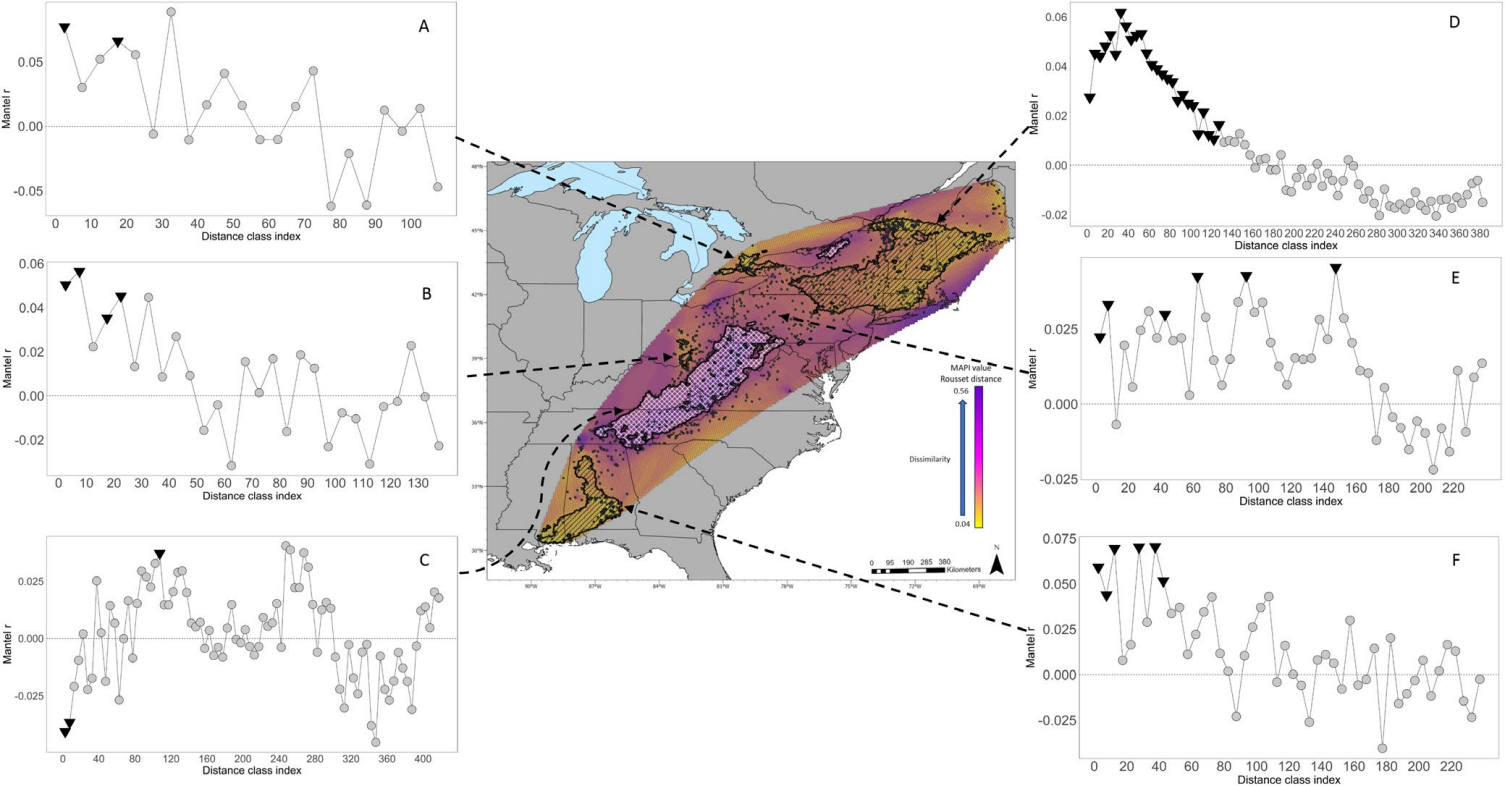
Mantel correlograms of raccoon genetic distances in 5 regions throughout the study area, which encompassed mainly (A) Ontario (B) Ohio and Kentucky, (C) Virginia, West Virginia, Pennsylvania, Alabama, Georgia, Kentucky, Maryland, Tennessee, Ohio, and North Carolina, (D) New York, Massachusetts, Maine Connecticut, New Hampshire, and Québec (E) Pennsylvania, Ohio, and New York, and (F) Alabama and Mississippi. The analysis was conducted on Rousset's distance *ȃ* versus geodesic distance. The *x*‐axis represents the distance class indexes for every 5 km (index is centered on the middle value of each class). The *y*‐axis is the Mantel *r* (correlation coefficient). Non‐significant values are represented by gray circles, and statistically significant values are inverted black triangles.

**FIGURE 7:**
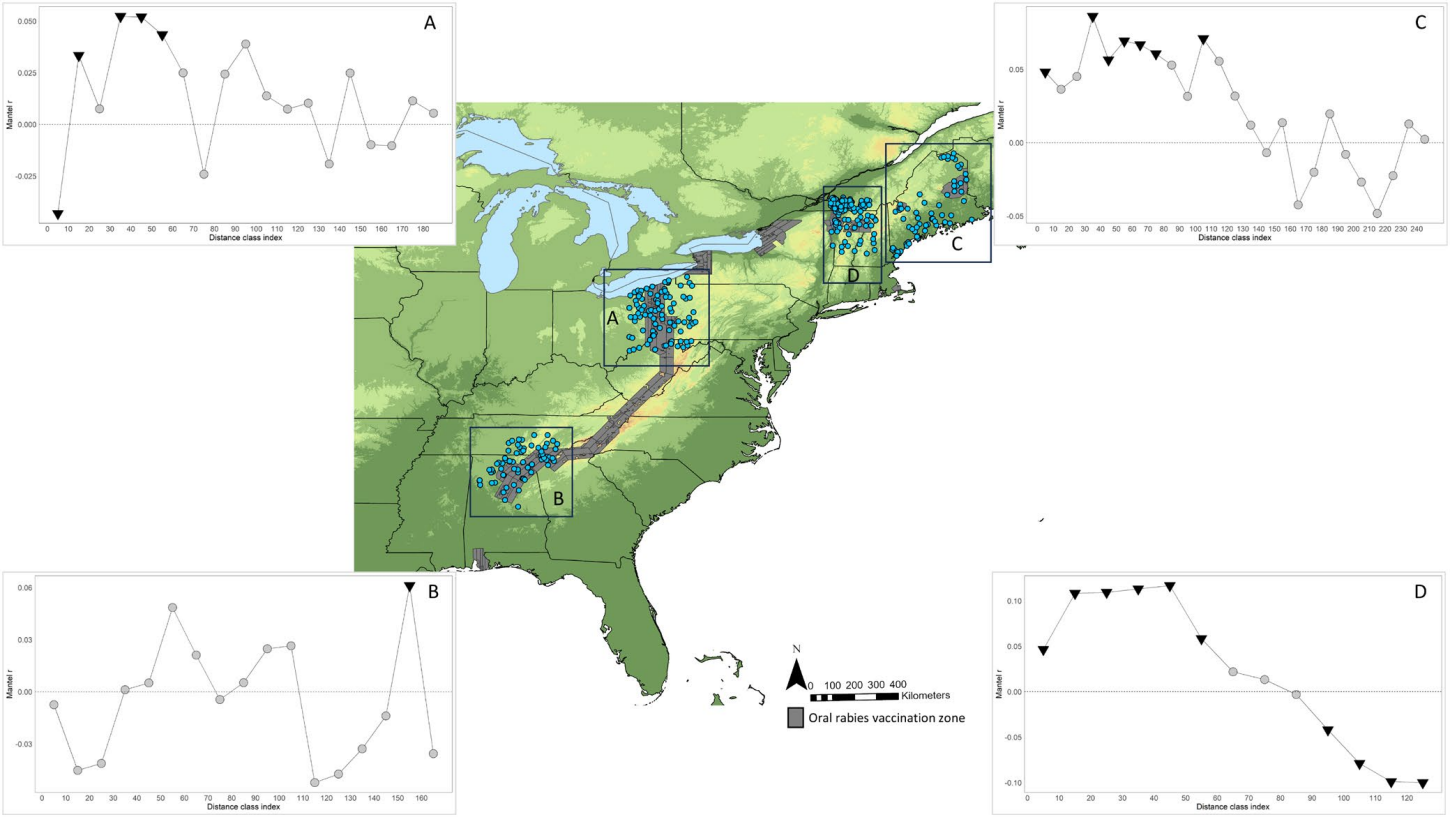
Mantel correlograms of raccoon genetic distances in 4 regions throughout the study area that had raccoon rabies emergency response actions in the last 10 years. The areas include (A) Ohio, Pennsylvania, and West Virginia, (B) Alabama, Georgia, and Tennessee, (C) Maine, (D) Vermont, New Hampshire, and Quebec. The analysis was conducted on Rousset's distance *ȃ* versus geodesic distance. The *x*‐axis represents the distance class indexes for every 10 km (index is centered on the middle value of each class). The *y*‐axis is the Mantel *r* (correlation coefficient). Non‐significant values are represented by gray circles, and statistically significant values are inverted black triangles. The dark gray regions on the map represent the oral rabies vaccination zone in 2023.

The mantel correlogram plots highlighted areas of both positive and negative spatial autocorrelation (Figures 5, 6, 7). Across the full dataset, positive spatial autocorrelation was significant until 160 km; then it dropped to zero (Figure 5). It was positive again at 180 km and became significant from 220 km to 410 km. It then declined to approximately zero before dropping to negative at 680 km. No additional distance classes were significant beyond 410 km.

Mantel correlograms from five regions representing high, low, and average spatial autocorrelation were compared to identify differences in local patterns of spatial autocorrelation (Figure 6). One group of samples represented an area of high connectivity identified using mapi, which included the northeastern states and Québec and had significant positive spatial autocorrelation out to 135 km (Figure 6, plot D). A large group of samples represented regions with high dissimilarity and encompassed the central and southern Appalachian Mountains in Virginia, West Virginia, Pennsylvania, Alabama, Georgia, Kentucky, Maryland, Tennessee, Ohio, and North Carolina (Figure 6, plot C). The Mantel correlogram demonstrated negative spatial autocorrelation out to 10 km, then increasing to positive at 40 km, which became significant at 110 km. Three geographic regions trended towards higher connectivity, which included: central Ohio and northern Kentucky (Figure 6, plot B); central and northern Pennsylvania, eastern Ohio, and southern New York (Figure 6, plot E); and southern and eastern Alabama (Figure 6, plot F). Central Pennsylvania showed a unique pattern of significant spatial autocorrelation out to 160 km that oscillated between significant and nonsignificant, then dropped to negative at 185 km (Figure 6, plot E). Ontario had a small region of high connectivity with significance that oscillated between positive and negative, starting at 25 km (Figure 6, plot A). Central Ohio had positive spatial autocorrelation out to 50 km (only out to 35 km was significant).

Spatial autocorrelation for the four regions that had rabies emergency responses in the last 10 years is presented in Figure 7. Plot A represents the Ohio/Pennsylvania region, where the first 10 km distance class showed significant negative spatial autocorrelation. Spatial autocorrelation was then positive and mostly significant out to 60 km, followed by fluctuations between negative and positive with no additional significant distance classes. Plot B represents the results from southeastern states with oscillating spatial autocorrelation and only a single positive significant data point at 160 km. Plot C shows positive spatial autocorrelation in Maine out to 140 km, but only significant out to 110 km. The final plot D presents spatial autocorrelation in Vermont/New Hampshire/Québec, which was positive and significant out to 60 km, then a rapid drop to significant negative autocorrelation at 100 km and beyond.

## 4 Discussion

Wide‐ranging, habitat generalist species can move through and occupy diverse landscapes, which can restrict population genetic differentiation. However, because all species have some level of limited dispersal ability and non‐random habitat selection behavior, subtle landscape population genetic structure that can be difficult to delineate (Frantz et al. 2009; Schwartz and Mckelvey 2009). Spatially explicit analyses combined with large genomic datasets can increase the resolution for detecting genetic clusters in broadly distributed species with low differentiation. We analyzed 1307 raccoon samples genotyped at 1000 microhaplotype loci from a large sampling area representing diverse habitat types in eastern North America. Multiple clusters of genetic ancestry that encompassed sizable geographic areas were evident, with zones of admixture between the clusters. We demonstrated localized population structure throughout the study area, and areas of high and low connectivity were likely influenced by the presence or absence of landscape features that impact raccoon movement. These results will pave the way for future studies on raccoons using landscape genetics to underscore ecoregion/habitat‐specific movement patterns that can inform management of the raccoon *Lyssavirus rabies* variant.

### 4.1 Spatial Genetic Patterns

Large‐scale raccoon population clusters were geographically structured throughout the study area. Interestingly, our results contrast with Cullingham, Kyle, et al. (2008), who reported negligible phylogeographic structure throughout the eastern US, with raccoon population haplotypes distributed across geographically disparate regions. The divergent results may be attributed to the different hypotheses (taxonomy versus population genetics), different molecular markers (mitochondrial versus nuclear), the number of loci (a single mitochondrial gene versus 1000 nuclear loci), and the geographic distribution of samples. However, an earlier study by Cullingham (2007) used a similarly distributed dataset and spatially explicit analyses to test phylogeographic hypotheses and found mtDNA clusters that were associated with specific regions. Similarities between the clusters in Cullingham (2007) and our results highlight two major origins of raccoon genetic ancestry in eastern North America: the southeastern US and the northeastern US and southeastern Canada.

Our structure analysis at *K* = 2 (largest *ΔK*) and the second eigenvector of the sPCA highlighted a contact zone between large clusters that encompassed the northeastern and southeastern regions. Genetic data from multiple North American animal species have revealed a phylogeographic break beginning around 40° latitude, which was the extent of the Laurentide ice sheet (Bernatchez and Wilson 1998; Delcourt 1981; Lyman and Edwards 2022; Soltis et al. 2006). We see two possible explanations for the patterns in northern and southern raccoon populations. As the glaciers retreated, southern raccoon populations may have expanded and colonized northern regions (Bernatchez and Wilson 1998). Alternately, raccoons may have persisted in multiple glacial refugia in the northern parts of the range, and when populations were reconnected, the isolated genetic diversity in each refugium mixed, resulting in the current geographic patterns, particularly in the central Appalachian region (Cullingham 2007; Lyman and Edwards 2022). Other small mammals in the northeast, such as grey squirrels ( *Sciurus carolinensis* ) and fishers ( *Martes pennanti* ), demonstrate similar population structure northeast of New York which implies that parallel biogeographical processes have impacted the evolution of multiple small mammalian species in this region (Fusco et al. 2024; Hapeman et al. 2011). Expanding the raccoon dataset to sample more of the genome and include more geographic regions may help partition the evolutionary impacts of Pleistocene, Holocene, and Anthropocene habitat fluctuations on raccoon historical biogeography.

As previously mentioned, raccoon populations sharply declined in some states in the 19th century due to overharvesting and habitat loss (Zeveloff 2002). We did not detect significantly low levels of genetic diversity in any of the genetic clusters, which suggests that raccoon effective population sizes have remained large enough throughout the last two centuries despite a period of decline. The relatively low *Fst* values, even across long distances, and the partition of most genetic variation within individuals, as identified by the AMOVA, suggest that genetic drift has not been a strong factor across the study area. In some species, historical signals of population structure are lost due to population bottlenecks, but given that genetic diversity and population structure of raccoons are similar to other co‐distributed small mammal species, this suggests raccoons have retained some signal of the historical biogeography (e.g., Fusco et al. 2024; Hapeman et al. 2011).

The Appalachian Mountain region was a significant driver of discontinuity in raccoon populations. The structure analyses revealed a cluster (K3) that overlaid the Appalachian Mountains (Figure 2). Spatial autocorrelation analyses demonstrated that the mountainous region had high genetic dissimilarity and negative spatial autocorrelation at distances less than 30 km, suggesting limited relatedness or gene flow among spatially proximate individuals (Figures 3, 5, and 6, plot B). Our data indicate that the mountain range has not been an absolute barrier to raccoon gene flow but likely restricts connectivity, possibly due to isolation‐by‐environment, where habitat patches drive genetic differentiation, even over short geographic distances (Wang and Bradburd 2014). Within upland pine‐deciduous forests, raccoons persist at lower population densities and have larger home ranges (Hill, Helton, Bernasconi, et al. 2023; Hill, Miller, Helton, et al. 2023; Slate et al. 2020). Puskas et al. (2010) found that raccoon groups may be constrained within valleys and have little contact with raccoons in neighboring valleys. Ultimately, the patchy habitats in mountainous regions and the resulting clumped resources likely lead to patterns in raccoon distribution, abundance, and social structure that are more sporadic or heterogeneous than regions with broadly distributed resources (Dharmarajan et al. 2014; Houle et al. 2011; Schuttler et al. 2015).

### 4.2 Implications for Rabies Management

Our discovery of regions in and around raccoon rabies management areas that harbor irregular spatial patterns of genetic diversity can provide useful information for the management of raccoon rabies in the eastern US. While it is not new information that raccoon ecology in higher elevation habitats is different from lowland areas, we have demonstrated that raccoons in the mountainous landscapes have limited contact with nearby raccoons and show minimal levels of connectivity with lowland raccoons. This is good news for wildlife rabies managers as the largest oral rabies vaccination (ORV) program integrates the Appalachian Mountains as a natural barrier to aid in stopping the westward expansion of raccoon rabies (Slate et al. 2005). An additional mountainous region to consider as a barrier is the Adirondacks in New York, which also appeared to restrict connectivity between raccoons in surrounding habitats (Figures 2, 3, 4). Further work to identify regions that have high or low genetic connectivity can inform the development of studies to test hypotheses about rabies virus movement, identify landscapes where there is a greater risk of pathogen spread, and formulate habitat‐specific management strategies (Biek and Real 2010; Kozakiewicz et al. 2018; Real and Biek 2007). However, one limitation of this study was the patchy distribution of samples from lowland areas east of the Appalachian Mountains in the southeastern US. The samples for this study were predominantly collected through raccoon rabies surveillance activities, which are focused in and directly west of the vaccination zones for detection of potential westward spread. We are currently working to rectify these sampling gaps for future studies.

In the southeastern US, there was a clear zone of spatial genetic cluster transition and admixture in northern Georgia, northern Alabama, and eastern Tennessee (K3 and K9, Figures 2 and 3). Raccoon connectivity appeared to be high in the southern Coastal Plain region of Alabama but limited in the northern part of the state. Raccoon rabies occurs in eastern Alabama, and an ORV zone established in northeastern Alabama helps prevent the westward spread of the rabies virus beyond the current management zone (Arjo et al. 2008). Interestingly, the virus has not established nor demonstrated significant movement through the central and southern Black Belt region of the state (Arjo et al. 2008), reducing the need to immediately establish an ORV zone in that area (Ma et al. 2024; Slate et al. 2005). We currently do not know what unique landscape characteristics in central and southern Alabama directly influence raccoon connectivity; models have estimated that soils (and the likely associated land cover) are a predictor of raccoon movement patterns in Alabama (Algeo et al. 2017). Expanding upon the current study to investigate both landscape genetics and landscape ecology can help further understand the mechanisms limiting raccoon rabies in Alabama to inform adaptive management of the pathogen (Rioux Paquette et al. 2014).

Throughout raccoon rabies management areas, specifically where emergency response actions have been implemented, population structure patterns were variable (Figure 7). The samples from Vermont/New Hampshire/Québec revealed short‐distance spatial autocorrelation that quickly declined to negative spatial autocorrelation (Figure 7; Plot D). This represents a strong pattern of IBD throughout this region with localized gene flow at distances less than 60 km and limited long‐distance dispersal. Beyond 60 km, the sharp transition from positive to negative points to factors other than distance as limiting gene flow. Vermont, New Hampshire, and Québec have diverse landscapes, ranging from mountains to the Champlain Valley, and landscape genetics approaches may help identify which habitats facilitate connectivity, informing targeted surveillance and vaccination. In Maine, localized gene flow occurs at distances less than 110 km with limited population structure beyond (Figure 7; plot C). Maine is a large state with expansive tracts of upland boreal forests. In this suboptimal raccoon habitat, populations are typically dispersed with low densities, which could explain the pattern of IBD to the longer distance of 110 km (Hill, Helton, Bernasconi, et al. 2023; Rosatte et al. 2010). The Mantel correlogram for Ohio/Pennsylvania highlights an interesting pattern where there is significant negative spatial autocorrelation at the 10 km distance class, and then IBD out to 60 km (Figure 7; plot A). The short‐distance negative autocorrelation could be due to a sampling artifact, or some behavioral/ecological dynamics (e.g., territoriality or high population turnover) in this area that allow for close association of genetically disparate individuals while still maintaining local population structure. In the final analysis, Alabama/Tennessee/Georgia (Figure 7; plot B) presents a case of likely high gene flow and genetic mixing across long distances. The lack of spatial autocorrelation at short distances can originate from high gene flow, long‐distance movements, or recent colonization/expansion. The single significant positive autocorrelation at 160 km suggests that genetically similar individuals occur at long distances, which could be due to long‐distance dispersal or translocations. This is a great concern for rabies management as the data suggest the rabies virus can move long distances quite rapidly in the southeastern US.

Across the spatial autocorrelation analyses, there were variable signals of positive spatial autocorrelation. These estimates suggest that in many regions, raccoon populations have a genetic influence that stretches out to at least 25 km, in some cases over 100 km in the northeastern states and Québec. Multiple studies have identified raccoon dispersal, predominantly males, that occasionally travel over 40 km (Cullingham, Pond, et al. 2008; Rosatte et al. 2010). The indirect connectivity estimates that we provide here complement these studies to suggest that when the raccoon variant of rabies virus is detected in certain regions, it could rapidly travel 25 km, perhaps even over 100 km from the point of detection. Additionally, when a rabid animal is found in a raccoon rabies‐free area, the autocorrelation distances can inform emergency response through guidance on the geographic scope for enhanced surveillance and emergency vaccination activities (Davis et al. 2024, 2023).

Population genetic structure of wildlife disease reservoirs is impacted by a balance of mutation, gene flow, genetic drift, and natural selection and can have profound effects on local adaptation, particularly in relation to pathogen resistance and maintenance (Cross et al. 2009; Gompper et al. 2011; Kyle et al. 2014). The field of genomics continues to develop ways to sample larger portions of an animal's genome for many animals at reasonable costs, which expands the ecological and evolutionary hypotheses we can test. Utilizing this technology and building research networks of geneticists, computational biologists, modelers, and wildlife disease managers can lead to highly rigorous research studies that reveal more aspects of a species' biology, which can facilitate improved management strategies and help limit the risk of pathogen transmission and spillover.

## Conflicts of Interest

The authors declare no conflicts of interest.

## Supporting information


Data S1.

## Data Availability

Data for this study are available through the NCBI Sequence Read Archive under accession numbers PRJNA950910 and PRJNA1149082.
