# Supplementary material for: Contrasting Patterns of Raccoon (Procyon lotor) Spatial Population Genomics Throughout a Rabies Management Area in Eastern North America
Source: Evol Appl. 2025 May 12;18(5):e70105. doi: 10.1111/eva.70105 (PMC12069805; doi:10.1111/eva.70105)

Figure S1. Results from structure *K* estimators for raccoons in eastern North America. The first plot is the mean LnP(*K*), the second is delta *K*, and the last is the four Puechamaille estimators.


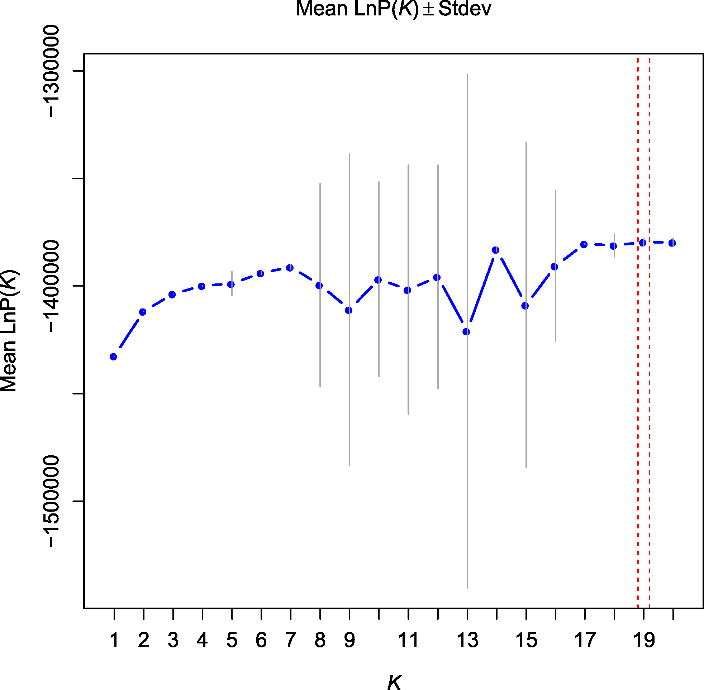


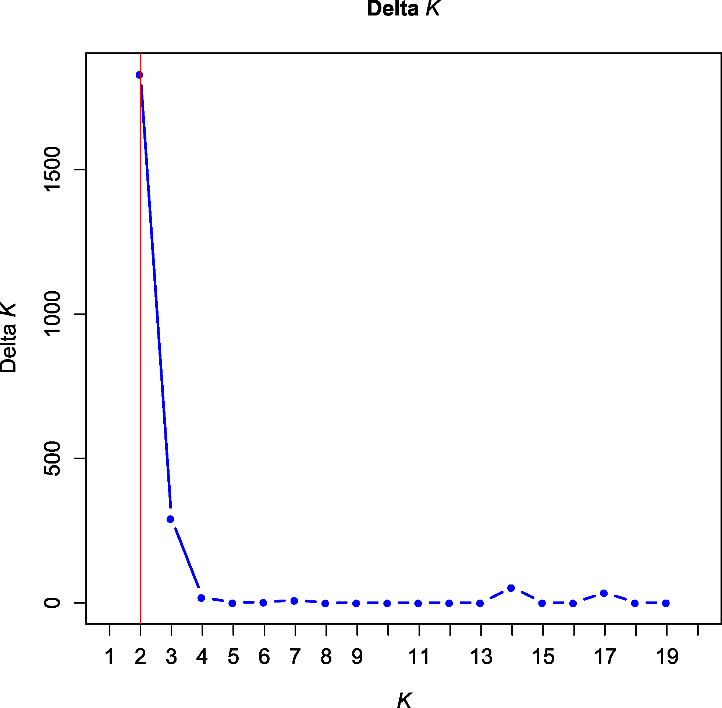


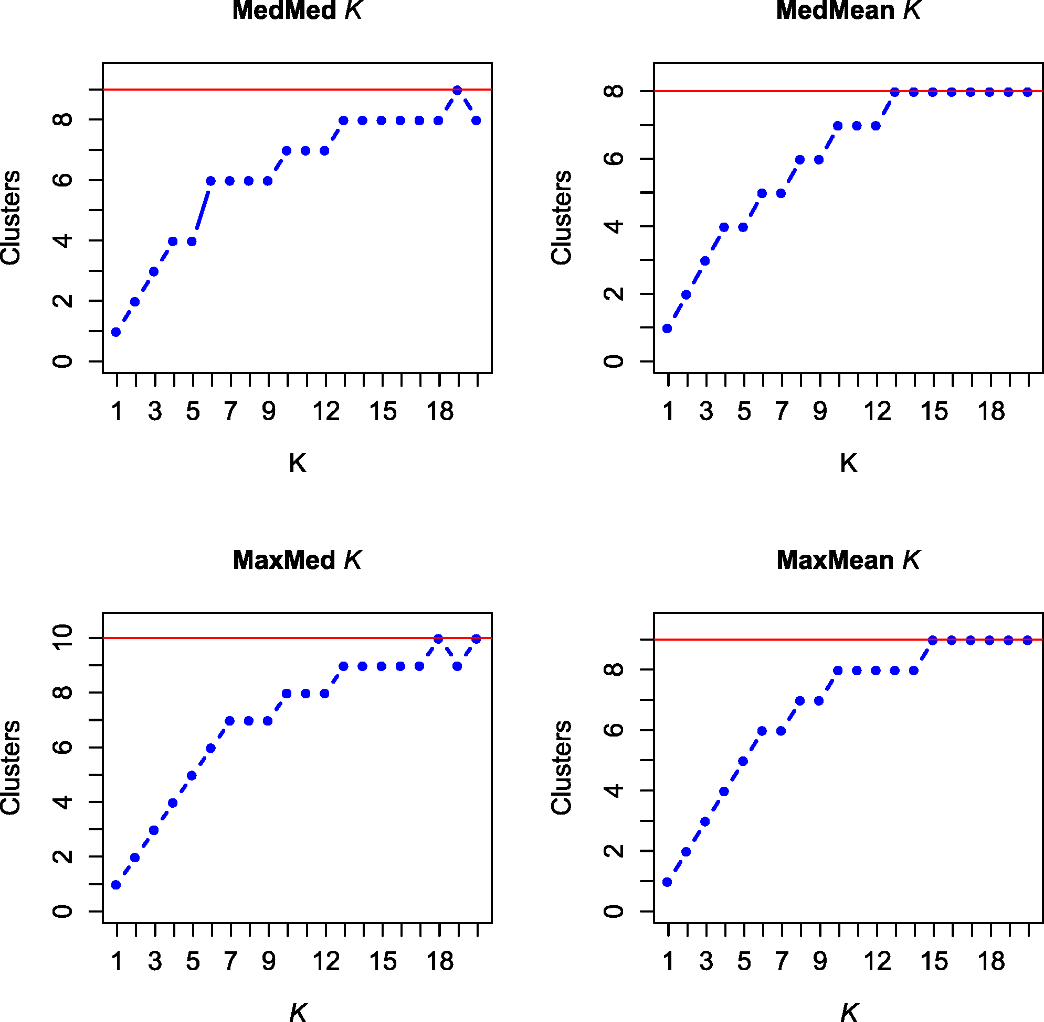


Figure S2. Genetic clustering results from the program structure at *K* = 2 for raccoons from eastern North America. Each pie chart represents an individual raccoon. Pie chart colors represent assignment to a genetic cluster.


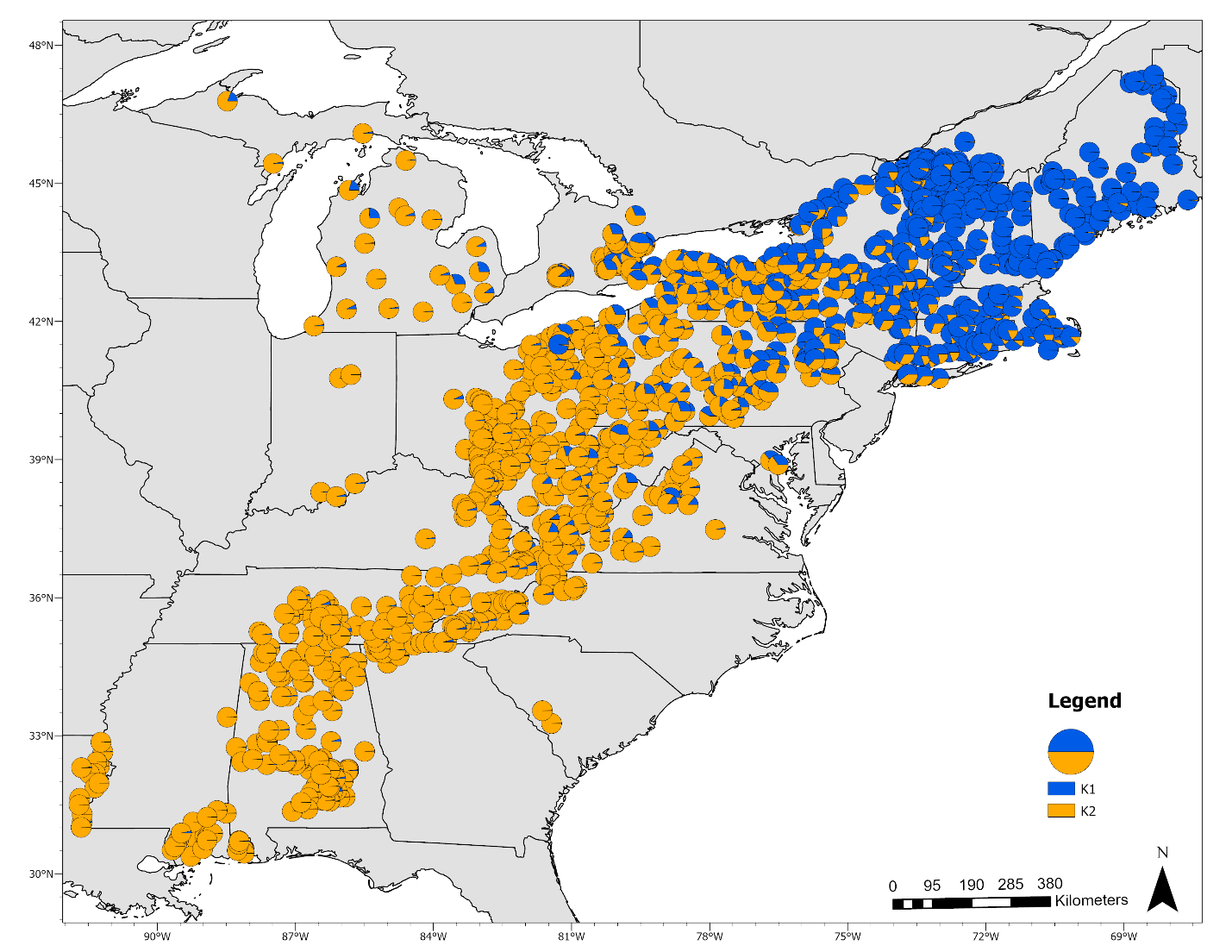


Figure S3. Genetic clustering results from the program structure at *K* = 3 for raccoons from eastern North America. Each pie chart represents an individual raccoon. Pie chart colors represent assignment to a genetic cluster.


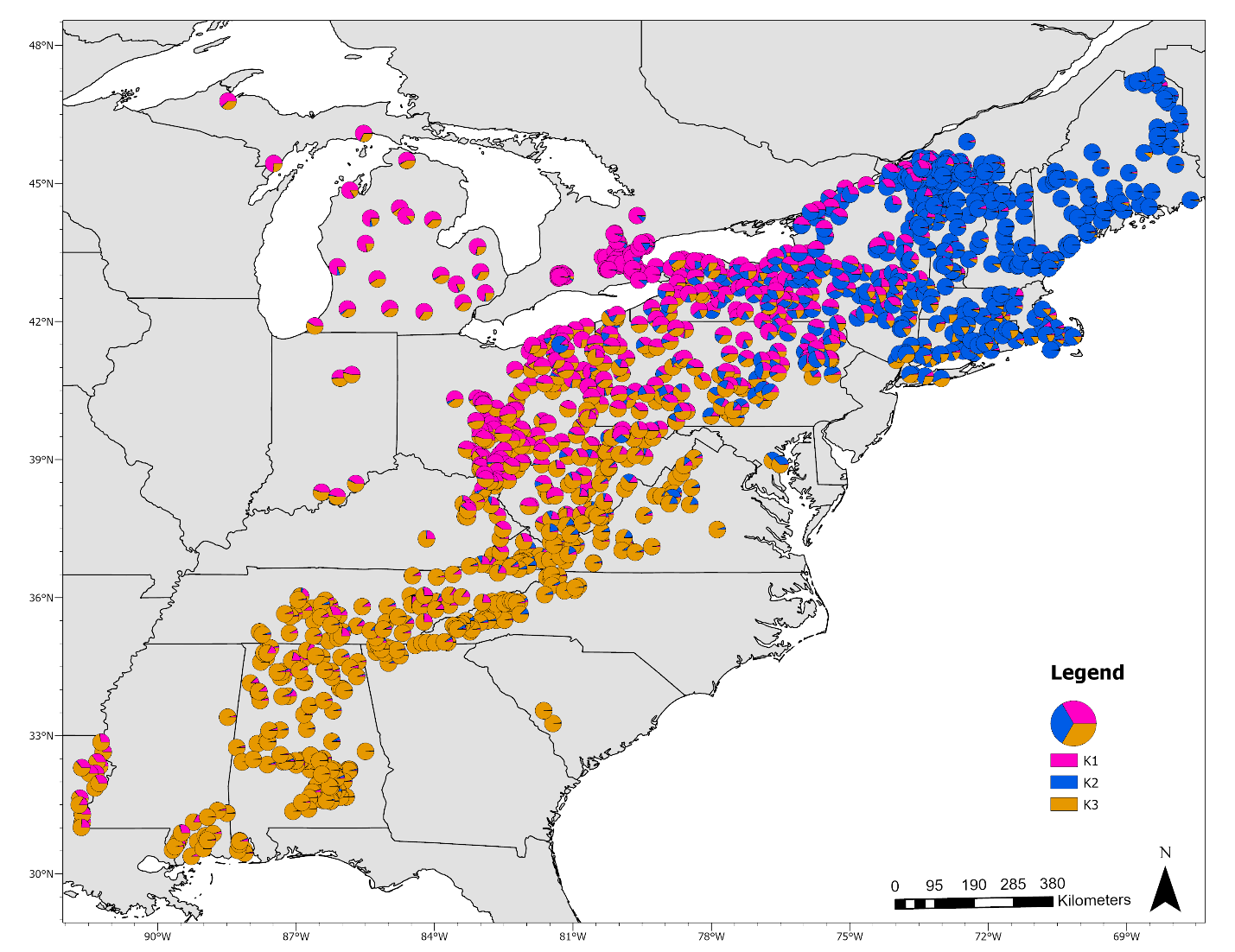


Figure S4. Scree plot of eigenvectors from spatial principal components for raccoons in eastern North America.


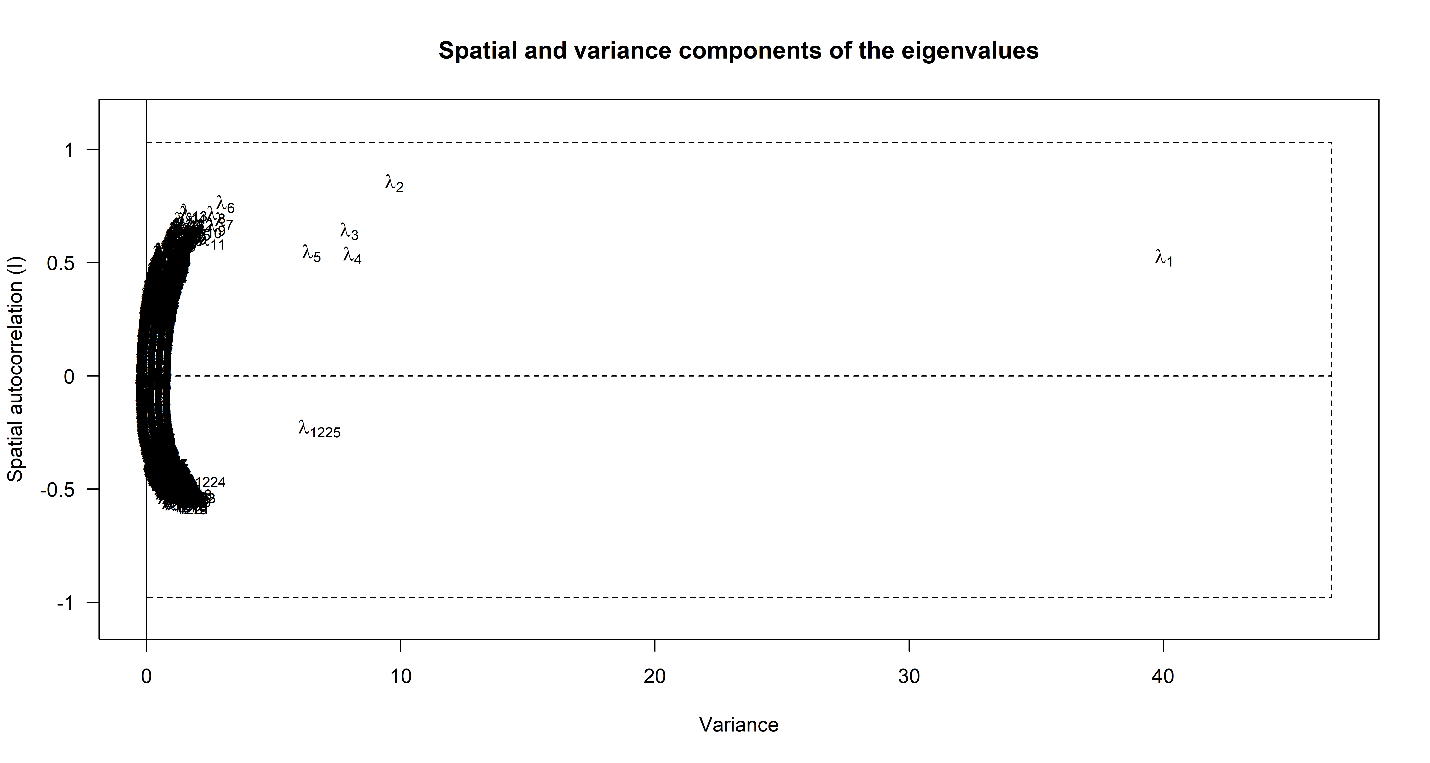

Supplement: Supplementary file 1 — Data S1. [file EVA-18-e70105-s001.docx]
